# Health Technology Readiness Profiles Among Danish Individuals With Type 2 Diabetes: Cross-Sectional Study

**DOI:** 10.2196/21195

**Published:** 2020-09-15

**Authors:** Ida Kær Thorsen, Sine Rossen, Charlotte Glümer, Julie Midtgaard, Mathias Ried-Larsen, Lars Kayser

**Affiliations:** 1 The Centre for Physical Activity Research University of Copenhagen Copenhagen Denmark; 2 Copenhagen Centre for Cancer and Health Municipality of Copenhagen Copenhagen Denmark; 3 Center for Diabetes Municipality of Copenhagen Copenhagen Denmark; 4 The University Hospitals' Centre for Health Research Rigshospitalet Copenhagen Denmark; 5 Department of Public Health University of Copenhagen Copenhagen Denmark

**Keywords:** readiness for health technology, telemedicine, diabetes mellitus, type 2, socioeconomic factors, mental health, psychological distress, healthcare disparities, delivery of healthcare, exercise

## Abstract

**Background:**

Information technologies (IT) are increasingly implemented in type 2 diabetes (T2D) treatment as a resource for remotely supported health care. However, possible pitfalls of introducing IT in health care are generally overlooked. Specifically, the effectiveness of IT to improve health care may depend on the user’s readiness for health technology.

**Objective:**

We aim to investigate readiness for health technology in relation to mental well-being, sociodemographic, and disease-related characteristics among individuals with T2D.

**Methods:**

Individuals with T2D (aged ≥18 years) who had been referred to self-management education, exercise, diet counseling, smoking cessation, or alcohol counseling completed a questionnaire survey covering (1) background information, (2) the 5-item World Health Organization Well-Being Index (WHO-5), (3) receptiveness to IT use in physical activity, and (4) the Readiness and Enablement Index for Health Technology (READHY), constituted by dimensions related to self-management, social support, and eHealth literacy. Individuals were divided into profiles using cluster analysis based on their READHY scores. Outcomes included differences across profiles in mental well-being, sociodemographic, and disease-related characteristics.

**Results:**

Participants in the study were 155 individuals with T2D with a mean age of 60.2 (SD 10.7) years, 55.5% (86/155) of which were men and 44.5% (69/155) of which were women. Participants were stratified into 5 health technology readiness profiles based on the cluster analysis: Profile 1, high health technology readiness; Profile 2, medium health technology readiness; Profile 3, medium health technology readiness and high level of emotional distress; Profile 4, medium health technology readiness and low-to-medium eHealth literacy; Profile 5, low health technology readiness. No differences in sociodemographic and disease-related characteristics were observed across profiles; however, we identified 3 vulnerable subgroups of individuals: Profile 3 (21/155, 13.5%), younger individuals (mean age of 53.4 years, SD 8.9 years) with low mental well-being (mean 42.7, SD 14.7) and emotional distress (mean 1.69, SD 0.38); Profile 4 (20/155, 12.9%), older individuals (mean age 66.3 years, SD 9.0 years) with less IT use (50.0% used IT for communication) and low-to-medium eHealth literacy; and Profile 5 (36/155, 23.2%) with low mental well-being (mean 43.4, SD 20.1) and low readiness for health technology.

**Conclusions:**

Implementation of IT in health care of individuals with T2D should be based on comprehensive consideration of mental well-being, emotional distress, and readiness for health technology rather than sociodemographic and disease-related characteristics to identify the individuals in need of social support, self-management education, and extensive IT support. A one-size-fits-all approach to IT implementation in health care will potentially increase the risk of treatment failure among the most vulnerable individuals.

## Introduction

Information technologies (IT) are increasingly implemented in type 2 diabetes (T2D) treatment throughout the health care system [1-3]. This stems from a common consensus that the implementation of IT, such as telehealth, health apps, social media, and the use of computers, smartphones, smartwatches, and tablets, has great potential for improving health care and self-management. Self-management is a crucial and ubiquitous element of T2D treatment [1]. In this context, digitalization is expected to promote the individual’s engagement in their own disease and health [2,3]. It is commonly held that digitalization strengthens the health professional–patient relationship, provides remote support, and increases time- and cost-efficiency [1-3]. Moreover, digitalization may facilitate the increasing person-centered and person-driven approach of health care, placing individuals in control of their own disease and treatment [2]. The use of IT is increasing among older populations (such as those who are ≥ 50 years), providing an easily accessible resource for remotely supported health care of individuals with T2D [4].

However, the possible pitfalls of introducing IT in health care have received little attention. Digitally supported weight loss or physical activity (PA) interventions have shown unexpected negative or lacking effects [5,6]. The effectiveness of IT implementation to improve health care and facilitate lifestyle change greatly depends on the user’s competencies, motivation, and experience with IT solutions [7]. In addition, the phenomenon known as the digital divide may further affect the potential of IT implementation to improve health care universally [8]. As such, individuals with T2D with increased age, low education levels, and of certain ethnic minority groups are more likely to lack access to IT solutions [8]. Low health literacy is common among these individuals and is independently associated with poor glycemic control [9]. Poor glycemic control and low education levels are further associated with the increased occurrence of depression symptoms, indicating a negative influence on mental well-being [10]. Additionally, low socioeconomic status is associated with nonattainment of T2D treatment goals [11] and is a strong predictor of all-cause and cardiovascular mortality [12]. Altogether, this gap in health care related to sociodemographic characteristics is at risk of widening if IT solutions are introduced in the health care of individuals with T2D without considering the individual´s readiness for health technology.

Readiness for health technology, including the user’s knowledge, skills, and attitudes towards health technology, their self-management of disease, and their social context, can be captured by the Readiness and Enablement Index for Health Technology (READHY) [13]. The READHY tool is based on a new understanding of eHealth literacy [14]. The eHealth Literacy Questionnaire (eHLQ) [15] constitutes the core of the tool, capturing the ability to seek, find, understand, and appraise health information from electronic sources and apply the knowledge gained to addressing or solving a health problem [15]. To further capture aspects of self-management and social support, this is supplemented by scales from 2 other validated tools [16,17].

The aims of the study are (1) to identify health technology readiness profiles among individuals with T2D using the READHY tool; (2) to investigate the differences between these profiles according to sociodemographic and disease-related characteristics, mental well-being, lifestyle factors, and IT use; and (3) to investigate the association of receptiveness to IT use in PA to sociodemographic and disease-related characteristics, mental well-being, smoking habits, and alcohol consumption.

## Methods

### Study Design, Setting, and Participants

This is a cross-sectional study conducted as a questionnaire survey, including background information and 3 instruments: (1) the 5-item World Health Organization Well-Being Index (WHO-5) [18], (2) receptiveness to IT use in PA [19], and (3) the READHY tool [13]. The questionnaire was administered on-site using paper and pencil and was partly interviewer- and self-administered with the possibility of receiving assistance.

**Figure 1:**
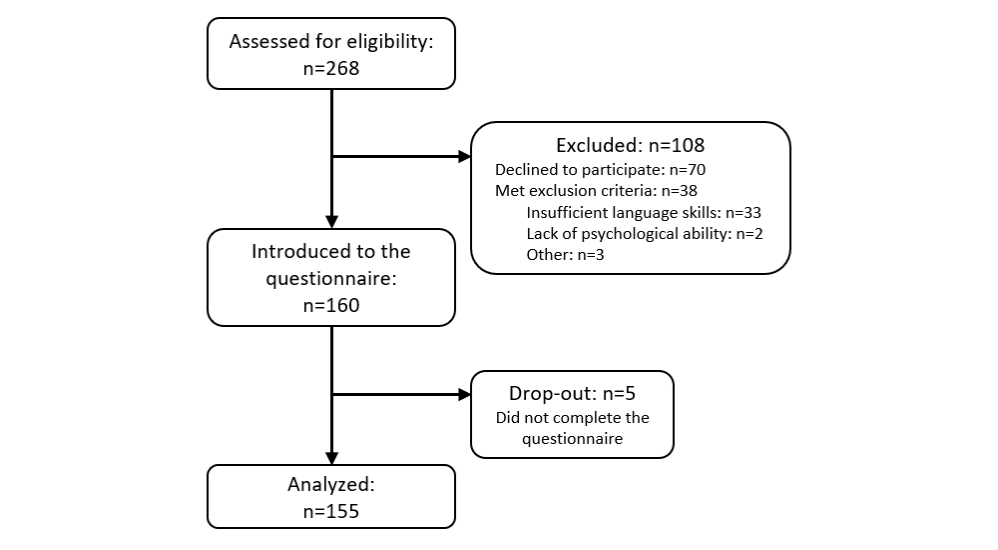
Flow of participants through the study (N=268).

Participants for the study were recruited directly from the Center for Diabetes, Municipality of Copenhagen in Denmark, which provides lifestyle programs for individuals with T2D such as self-management education, exercise, diet counseling, smoking cessation, or alcohol counseling. The participant flow is depicted in Figure 1. All the individuals (N=268) who had an appointment at the center during the time period of February 5, 2018, to March 28, 2018, were approached by a project staff member. Eligibility for participation was based on the inclusion criteria of being ≥18 years of age and having a T2D diagnosis, and exclusion criteria of insufficient Danish language skills and a lack of a psychological ability to answer questions as per the evaluation of a health care professional or project staff member. A total of 155 individuals were included in the survey, resulting in a response rate of 57.8%. The individuals that declined or met exclusion criteria had a mean age of 58.8 (SD 12.0) years, 56.6% (64/113) of which were men and 43.4% (49/113) of which were women. According to these parameters, they did not differ markedly from the included individuals. Participants provided oral and written consent prior to participation. The ethical committee of the Capital Region of Denmark confirmed that ethical approval was not required (18012824).

### Measures

#### Sociodemographic and Disease-Related Characteristics, Lifestyle Factors, and IT Use

Background information on sociodemographic and disease-related characteristics, lifestyle factors, and IT use were collected via self-report and include sex, age, education level, cohabitation status, source of income, time since diagnosis, type of medication, T2D complications, additional chronic conditions, smoking habits, alcohol consumption, daily PA, participation in lifestyle programs, and ownership and purpose of IT use. Education level was categorized according to the International Standard Classification of Education 2011 (ISCED-2011) [20] as follows: comprehensive school (ISCED-2011 levels 1–2), short education (ISCED-2011 levels 3–5), medium education (ISCED-2011 level 6), and long education (ISCED-2011 levels 7–8). Alcohol consumption was evaluated according to recommendations for men and women, respectively [21].

#### Mental Well-Being

Mental well-being was assessed using the WHO-5 [18]. Risk of depression was defined as scores of <50 [18].

#### The Readiness and Enablement Index for Health Technology (READHY)

The READHY tool was used to assess readiness for health technology. The READHY tool consists of the eHealth Literacy Questionnaire (eHLQ) [15] that includes 7 scales, supplemented with 4 scales from the Health Education Impact Questionnaire (heiQ) [17] and 2 scales from the Health Literacy Questionnaire (HLQ) [16]. Together, these scales capture eHealth literacy, self-management, and social context. The READHY tool has been validated [13]. The 13 scales were assessed using 65 items, each item presented to the participant as a statement and scored on a 4-point rating, from 1=strongly disagree to 4=strongly agree. The overall score of each scale was calculated as the mean score of the 4–6 items (ie, statements) that constitute the scale. If <50% of items in a scale were answered, the scale was regarded as missing and the questionnaire survey was considered incomplete for the respondent.

#### Receptiveness to IT Use in Physical Activity

Receptiveness to IT use in PA was categorized into 2 groups, receptive and nonreceptive, according to self-reported answers (“yes” or “no,” respectively) to the question, “Can you imagine supplementing exercise with the use of IT solutions?”

### Statistical Methods

The participants for this study constituted a convenience sample obtained during the period of February 5, 2018, to March 28, 2018.

Using a data driven approach, a combination of hierarchical and K-means cluster analysis was applied to the READHY data to divide participants into clusters (hereafter referred to as profiles) according to their level of readiness for health technology. Hierarchical cluster analysis with Ward’s method for linkage (L2 squared measure) was used to determine the optimal number of profiles. Evaluation of the dendrogram, elbow method, and silhouette coefficients assessed 4 profiles as the best fit and 5 profiles as the second-best fit.

Thereafter, K-means cluster analysis was conducted with both the 4- and 5-profile solution in 8 iterations. For both solutions, the difference between profiles for each READHY scale was assessed using a one-way analysis of variance (ANOVA). The magnitude of the F value indicates how well the respective scale discriminates between profiles. Pairwise comparisons were performed if indicative by the one-way ANOVA (*P*<.05). The READHY scale scores for each of the profiles are reported as a mean and standard deviation (SD). For both the 4- and 5-profile solution, differences between the identified profiles in sociodemographic and disease-related characteristics, mental well-being, lifestyle factors, and IT use were tested using the Fisher exact test for frequencies and using the one-way ANOVA for continuous variables. Pairwise comparisons were performed if indicative by the Fisher exact test or one-way ANOVA (*P*<.05). Frequencies are reported as numbers and proportions, and continuous variables are reported as a mean and SD. We found that the 5-profile solution contained the information from the 4-profile solution and further added information to the analyses. Therefore, we chose to only report the results from the 5-profile solution. The correlation between mental well-being and heiQ8-emotional distress was tested using the Pearson’s correlation to evaluate the association between these 2 parameters.

The association of sociodemographic and disease-related characteristics, mental well-being, smoking habits, and alcohol consumption to the receptiveness to IT use in PA were tested using logistic regression. Receptiveness to IT use in PA was defined as the binary outcome variable. All exposure variables were included in the model individually. For each of the exposure variables, the odds ratio and a 95% confidence interval for receptiveness to IT use in PA are reported. *P* values and 95% confidence intervals were calculated using exact statistics.

Model assumptions were investigated prior to analyses by investigating predicted values and standardized residuals. Data were analyzed as observed—no imputations were used to replace missing data. All statistical analyses were performed using Stata IC 13 (StataCorp). The significance level was set to *P*<.05 (2-tailed).

### Role of the Funding Source

The funding source was not involved in the study design, in the collection, analysis, or interpretation of data, in writing the report; or in the decision to submit the paper for publication. IKT had full access to all the data in the study. All authors had final responsibility for the decision to submit for publication.

## Results

### Participant Characteristics

Participants had a mean age of 60.2 (SD 10.7) years; 55.5% (86/155) were men and 44.5% (69/155) were women. Most participants were diagnosed less than 5 years ago (124/155, 80.0%), were prescribed peroral medication (102/155, 65.8%), and experienced T2D complications (90/122, 73.8%). Moreover, one-third of the participants were at risk of depression (49/154, 31.8%), and most participants had 2 or more additional chronic conditions (78/155, 50.3%), owned a smartphone (111/155, 71.6%), and reported a wish to be more physically active (125/155, 80.7%).

### Readiness for Health Technology

**Figure 2:**
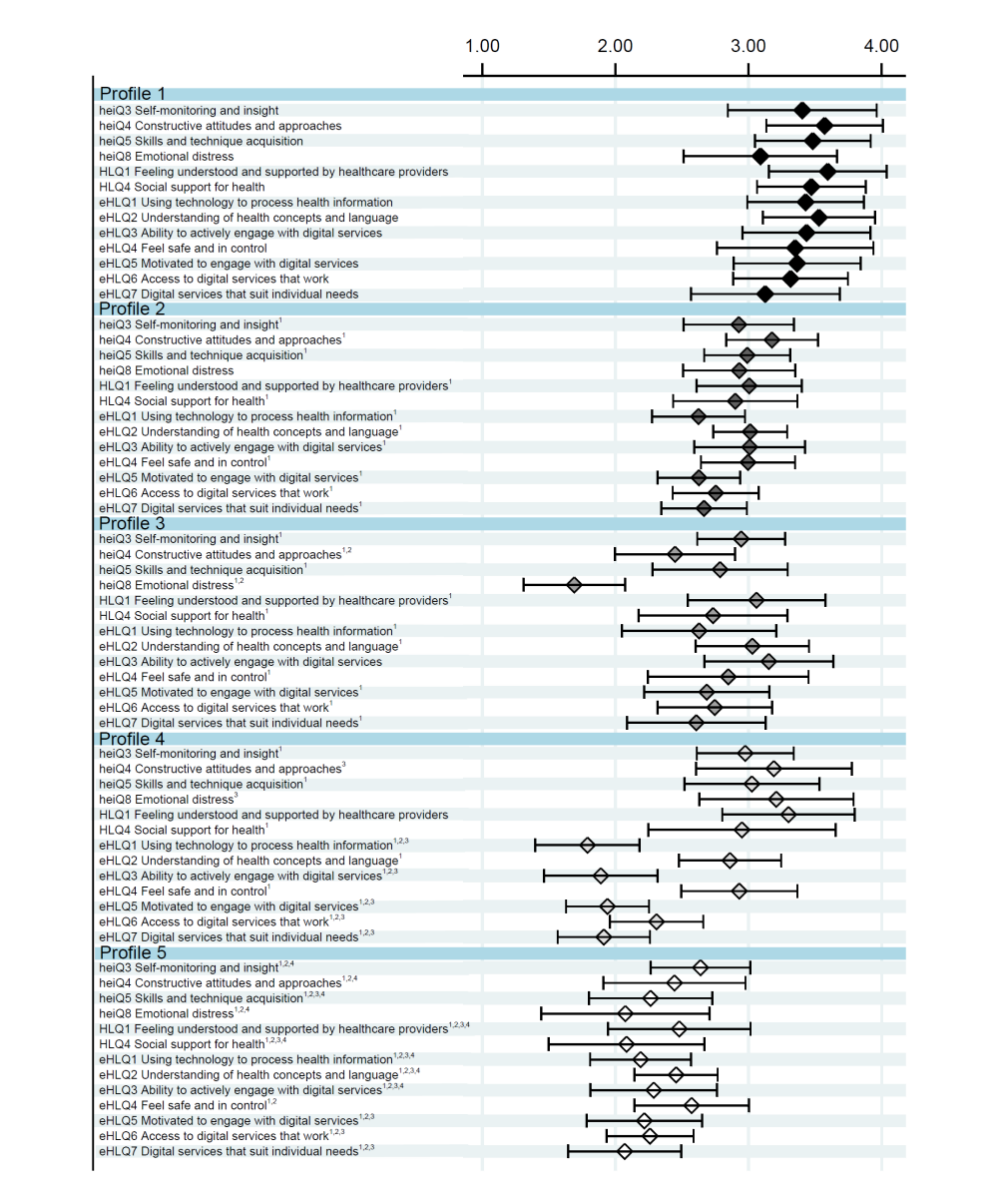
Readiness and Enablement Index for Health Technology (READHY) scale scores for the 5 identified profiles based on cluster analysis. heiQ: Health Education Impact Questionnaire; HLQ: Health Literacy Questionnaire; eHLQ: eHealth Literacy Questionnaire. Data are presented as mean (SD). heiQ8 was reversed (ie, a high score indicates a low level of emotional distress). heiQ3, F=13.16; heiQ4, F=34.38; heiQ5, F=33.81; heiQ8, F=42.87; HLQ1, F=24.24; HLQ4, F=28.12; eHLQ1, F=55.49; eHLQ2, F=37.90; eHLQ3, F=51.19; eHLQ4, F=11.31; eHLQ5, F=48.17; eHLQ6, F=39.12; eHLQ7, F=35.52.

The combined data-driven cluster analyses resulted in 5 distinct profiles within which participants were similar regarding readiness for health technology, with a large variability between profiles and a small variability within profiles (Figure 2). Profiles 1 to 5 are presented in ascending order according to their overall mean READHY score. Of the 5 profiles, 3 profiles consistently scored high, medium, and low, respectively, whereas 2 profiles had varying scores on some scales. Profile 1 (n=28) consistently scored high on all scales. Profile 2 (n=50) consistently scored medium on all scales. Profile 3 (n=21) scored medium on all scales, except for low scores on heiQ4-constructive attitudes and approaches and heiQ8-emotional distress. Profile 4 (n=20) generally scored high on scales related to self-management and social support and low-to-medium on scales related to eHealth literacy; Profile 5 (n=36) consistently scored low on all scales. Overall between-group differences were observed across profiles (*P*=.001). Pairwise comparisons of each scale are presented in Figure 2.

### Sociodemographic Characteristics

Sociodemographic characteristics are presented in Table 1. Participants in Profile 3 were younger than participants in Profiles 1 and 4 (-9.0 years, *P*=.03; and -12.8 years, *P*=.001, respectively). More participants in Profile 3 received public income support or had no income compared with participants in Profiles 1, 2, and 4 (+39.2 percent points (pp), *P*=.02, +45.1 pp, *P*=.001, and +47.1 pp, *P*=.004, respectively) of which more received retirement pension (+25.0 pp, *P*=.02, +23.4 pp, *P*=.001, and +36.4 pp, *P*=.004, respectively). More participants in Profile 2 received salary compared with participants in Profile 3 (+21.7 pp, *P*=.001). Moreover, more participants in Profile 5 received public income support or had no income compared with participants in Profiles 2 and 4 (+21.3 pp, *P*=.02 and +23.3 pp, *P*=.04, respectively) of which more received retirement pension (+15.9 pp *P*=.02, and +28.9 pp, *P*=.04, respectively). There were no differences across profiles in sex, highest level of education, or cohabitation status (*P*>.05).

**Table 1:** Sociodemographic characteristics of participants (N=155) across profiles [data are presented as mean (SD) for continuous variables and numbers (proportions) for frequencies].

Characteristics		All (N=155)		Profile 1 (n=28, 18.1%)		Profile 2 (n=50, 32.2%)		Profile 3 (n=21, 13.5%)		Profile 4 (n=20, 12.9%)		Profile 5 (n=36, 23.2%)		*P* value	
**Sex, n (%)**															.46
	Women		69 (44.5)		13 (46.4)		21 (42.0)		9 (42.9)		6 (30.0)		20 (55.6)		
	Men		86 (55.5)		15 (53.6)		29 (58.0)		12 (57.1)		14 (70.0)		16 (44.4)		
Age, mean (SD)		60.2 (10.7)		62.4 (10.6)		59.8 (11.3)		53.4 (8.9)^a^		66.3 (9.0)^b^		59.8 (9.6)		.002	
**Highest attained level of education, n (%)**															.15
	Comprehensive school		24 (15.5)		2 (7.2)		7 (14.0)		7 (33.3)		1 (5.0)		7 (19.4)		
	Short education		81 (52.2)		13 (46.4)		28 (56.0)		8 (38.1)		16 (80.0)		16 (44.5)		
	Medium education		33 (21.3)		10 (35.7)		9 (18.0)		4 (19.1)		1 (5.0)		9 (25.0)		
	Long education		17 (11.0)		3 (10.7)		6 (12.0)		2 (9.5)		2 (10.0)		4 (11.1)		
**Cohabitation status, n (%)**															.69
	Living alone		78 (50.3)		13 (46.4)		22 (44.0)		12 (57.1)		10 (50.0)		21 (58.3)		
	Living with spouse and/or children		77 (49.7)		15 (53.6)		28 (56.0)		9 (42.9)		10 (50.0)		15 (41.7)		
**Source of income, n (%)**															.007
	Salary		45 (29.0)		8 (28.6)		18 (36.0)		3 (14.3)^c^		5 (25.0)		11 (50.6)		
	Retirement pension		73 (47.1)		15 (53.6)		26 (52.0)		6 (28.6)^a,c^		13 (65.0)^b^		13 (36.1)^c,d^		
	Public income support/no incomes		37 (23.9)		5 (17.8)		6 (12.0)		12 (57.1)^a,c^		2 (10.0)^b^		12 (33.3)^c,d^		

^a^Different from Profile 1, *P*<.05.

^b^Different from Profile 3, *P*<.05.

^c^Different from Profile 2, *P*<.05.

^d^Different from Profile 4, *P*<.05.

### Disease-Related Characteristics and Mental Well-being

Disease-related characteristics and mental well-being are presented in Table 2. Participants in Profiles 3 and 5 scored lower on mental well-being compared to participants in Profiles 1, 2, and 4 (Profile 3, -29.0, -24.6, and -24.5, respectively, *P*=.001; Profile 5, -23.8, -23.8, and -23.8, respectively, *P*=.001). Further, more participants in Profile 3 and 5 were at risk of depression compared with participants in Profiles 1, 2, and 4 (Profile 3, +56.0 pp, +54.5 pp, and +56.7 pp, respectively, *P*=.001; Profile 5, +56.0 pp, +54.5 pp, and +56.7 pp, respectively, *P*=.001). The correlation between mental well-being and heiQ8-emotional distress was strong (r=0.645, *P*=.001). There were no differences across profiles in time since diagnosis, type of medication, T2D complications, and additional chronic conditions (*P*>.05).

**Table 2:** Disease-related characteristics and mental well-being of participants (N=155) across profiles [data are presented as mean (SD) for continuous variables and numbers (proportions) for frequencies].

Characteristics		All (N=155)	Profile 1 (n=28, 18.1%)	Profile 2 (n=50, 32.3%)	Profile 3 (n=21, 13.5%)	Profile 4 (n=20, 12.9%)	Profile 5 (n=36, 23.2%)		*P* value
**Time since diabetes diagnosis, n (%)**								.87	
	≤5 years	124 (80.0)	22 (78.6)	42 (82.0)	16 (76.2)	15 (75.0)	29 (80.6)		
	≥6 years	31 (20.0)	6 (21.4)	8 (16.0)	5 (23.8)	5 (25.0)	7 (19.4)		
**Type of medication, n (%)**								.58	
	None	19 (12.3)	4 (14.3)	8 (16.0)	3 (14.3)	1 (5.0)	3 (8.3)		
	Peroral	102 (65.8)	19 (67.9)	32 (64.0)	10 (47.6)	16 (80.0)	25 (69.5)		
	Injection	34 (21.9)	5 (17.8)	10 (20.0)	8 (38.1)	3 (15.0)	8 (22.2)		
**T2D complications, n (%)^a^**								.37	
	Yes	90 (73.8)	5 (22.7)	7 (18.0)	6 (35.3)	7 (41.2)	7 (25.9)		
	No	32 (26.2)	17 (77.3)	32 (82.0)	11 (64.7)	10 (58.8)	20 (74.1)		
**Additional chronic conditions, n (%)**								.05	
	No additional conditions	17 (11.0)	3 (10.7)	10 (20.0)	1 (4.8)	2 (10.0)	1 (2.8)		
	1 additional condition	60 (38.7)	13 (46.4)	23 (46.0)	5 (23.8)	7 (35.0)	12 (33.3)		
	2+ additional conditions	78 (50.3)	12 (42.9)	17 (34.0)	15 (71.4)	11 (55.0)	23 (63.9)		
Mental well-being, mean (SD)^b^		59.1 (20.2)	71.7 (16.3)	67.3 (14.6)	42.7 (14.7)^c,d^	67.2 (12.7)^e^	43.4 (20.1)^c,d,f^		<.001
Risk of depression (score <50), n (%)^b^		49 (31.8)	3 (10.7)	6 (12.2)	14 (66.7)^c,d^	2 (10.0) ^e^	24 (66.7)^c,d,f^		.001

^a^n=122.

^b^n=154.

^c^Different from Profile 1, *P*<.05.

^d^Different from Profile 2, *P*<.05.

^e^Different from Profile 3, *P*<.05.

^f^Different from Profile 4, *P*<.05.

### Lifestyle Factors

Lifestyle factors are presented in Table 3. There were no differences across profiles in smoking habits, alcohol consumption, daily PA, wish to be more physically active, change in exercise habits with T2D, or participation in lifestyle or exercise programs (*P*>.05).

**Table 3:** Lifestyle factors of participants (N=155) across profiles [data are presented as numbers (proportions)].

Lifestyle factors		All (N= 155)	Profile 1 (n=28, 18.1%)	Profile 2 (n=50, 32.3%)	Profile 3 (n=21, 13.5%)	Profile 4 (n=20, 12.9%)	Profile 5 (n=36, 23.2%)	*P* value
**Smoking habits, n (%)^a^**								.82
	Current	26 (16.9)	2 (7.1)	8 (16.3)	6 (28.6)	3 (15.0)	7 (19.4)	
	Earlier	76 (49.3)	16 (57.2)	23 (47.0)	9 (42.8)	10 (50.0)	18 (50.0)	
	Never	52 (33.8)	10 (35.7)	18 (36.7)	6 (28.6)	7 (35.0)	11 (30.6)	
**Alcohol consumption, n (%)^a^**								.08
	No alcohol	55 (35.7)	9 (32.1)	11 (22.4)	11 (52.4)	5 (25.0)	19 (52.8)	
	According to recommendations	85 (55.2)	16 (57.2)	34 (69.4)	9 (42.8)	13 (65.0)	13 (36.1)	
	Above recommendations	14 (9.1)	3 (10.7)	4 (8.2)	1 (4.8)	2 (10.0)	4 (11.1)	
**Daily physical activity, n (%)^a^**								.77
	<30 min/day	38 (24.7)	5 (17.9)	14 (28.6)	3 (14.3)	6 (30.0)	10 (27.8)	
	30-60 min/day	69 (44.8)	14 (50.0)	19 (38.8)	13 (61.9)	7 (35.0)	16 (44.4)	
	>60 min/day	47 (30.5)	9 (32.1)	16 (32.6)	5 (23.8)	7 (35.0)	10 (27.8)	
**Wish to be more physically active, n (%)**								.42
	Yes	125 (80.7)	23 (82.2)	37 (74.0)	21 (100.0)	16 (80.0)	28 (77.8)	
	No	12 (7.7)	2 (7.1)	6 (12.0)	0 (0.0)	2 (10.0)	2 (5.5)	
	Maybe	18 (11.6)	3 (10.7)	7 (14.0)	0 (0.0)	2 (10.0)	6 (16.7)	
**Change in exercise habits with T2D, n (%)**								.14
	Increased	75 (48.4)	17 (60.7)	25 (50.0)	12 (57.1)	7 (35.0)	14 (38.9)	
	Unchanged	73 (47.1)	10 (35.7)	24 (48.0)	6 (28.6)	12 (60.0)	21 (58.3)	
	Decreased	7 (4.5)	1 (3.6)	1 (2.0)	3 (14.3)	1 (5.0)	1 (2.8)	
**Lifestyle intervention, n (%)**								.28
	No lifestyle courses	41 (26.4)	10 (35.7)	12 (24.0)	6 (28.6)	2 (10.0)	11 (30.6)	
	1 lifestyle course	35 (22.6)	3 (10.7)	10 (20.0)	5 (23.8)	9 (45.0)	8 (22.2)	
	2+ lifestyle courses	79 (51.0)	15 (53.6)	28 (56.0)	10 (47.6)	9 (45.0)	17 (47.2)	
**Exercise intervention, n (%)**								.94
	Yes	73 (47.1)	13 (46.4)	25 (50.0)	9 (42.9)	8 (40.0)	18 (50.0)	
	No	82 (52.9)	15 (53.6)	25 (50.0)	12 (57.1)	12 (60.0)	18 (50.0)	

^a^n=154

### IT Use

Factors related to IT use are presented in Table 4. Fewer participants in Profile 4 owned a smartphone compared to participants in Profiles 1 and 2 (-33.6 pp, *P*=.03; and -37.0 pp, *P*=.003, respectively). Compared with participants in Profiles 1, 2, and 3, fewer participants in Profile 4 used IT for information seeking (-27.9 pp, *P*=.02, -29.0 pp, *P*=.004, and -30.2 pp, *P*=.02, respectively), communication with family and friends (-42.9 pp, *P*=.002, -42.0 pp, *P*=.001, and -45.2 pp, *P*=.001, respectively), and entertainment (-39.3 pp, *P*=.004, -26.0 pp, *P*=.05, and -45.2 pp, *P*=.001, respectively). Moreover, fewer participants in Profile 5 used IT for entertainment compared to participants in Profiles 1 and 3 (-23.6 pp, *P*=.04, and -29.5 pp, *P*=.02, respectively). Finally, fewer participants in Profile 4 were receptive to IT use in PA compared to participants in Profiles 1, 2, and 3 (-38.6 pp, *P*=.01, -34.0 pp, *P*=.01, and -41.0 pp, *P*=.01, respectively). There were no differences across profiles in smartwatch, tablet, and computer ownership, or exercise and work purposes of IT use (*P*>.05).

**Table 4:** Information technologies (IT) use of participants (N=155) across profiles [data are presented as numbers (proportions)].

IT use		All (N=155)	Profile 1 (n=28, 18.1%)	Profile 2 (n=50, 32.3%)	Profile 3 (n=21, 13.5%)	Profile 4 (n=20, 12.9%)	Profile 5 (n=36, 23.2%)	*P* value	
**Owns smartphone, n (%)**									.03
	Yes	111 (71.6)	22 (78.6)	41 (82.0)	16 (76.2)	9 (45.0)^a,b^	23 (63.9)		
	No	44 (28.4)	6 (21.4)	9 (18.0)	5 (23.8)	11 (55.0)^a,b^	13 (36.1)		
**Owns smartwatch, n (%)**									>.99
	Yes	8 (5.2)	1 (3.6)	3 (6.0)	1 (4.8)	1 (5.0)	2 (5.6)		
	No	147 (94.8)	27 (96.4)	47 (94.0)	20 (95.2)	19 (95.0)	34 (94.4)		
**Owns tablet, n (%)**									.12
	Yes	82 (52.9)	9 (32.1)	30 (60.0)	10 (47.6)	7 (35.0)	16 (44.4)		
	No	73 (47.1)	19 (67.9)	20 (40.0)	11 (52.4)	13 (65.0)	20 (55.6)		
**Owns computer (NOT smartphone, smartwatch, or tablet), n (%)**									.08
	Yes	24 (15.5)	2 (7.1)	5 (10.0)	3 (14.3)	7 (35.0)	7 (19.4)		
	No	131 (84.5)	26 (92.9)	45 (90.0)	18 (85.7)	13 (65.0)	29 (80.6)		
**Purpose of using IT, n (%)^c^**									
	Exercise	27 (17.5)	7 (25.0)	9 (18.0)	2 (9.5)	2 (10.0)	7 (20.0)	.61	
	Work	55 (35.7)	10 (35.7)	21 (42.0)	5 (23.8)	6 (30.0)	13 (37.1)	.67	
	Information seeking	135 (87.7)	26 (92.9)	47 (94.0)	20 (95.2)	13 (65.0)^a,b,d^	29 (82.9)	.01	
	Communication with family/friends	129 (83.8)	26 (92.9)	46 (92.0)	20 (95.2)	10 (50.0)^a,b,d^	27 (77.1)	.001	
	Entertainment	116 (75.3)	25 (89.3)	38 (76.0)	20 (95.2)	10 (50.0)^a,b,d^	23 (65.7)^a,d^	.003	
**Receptiveness to IT use in PA, n (%)**									.03
	Receptive	107 (69.0)	22 (78.6)	37 (74.0)	17 (81.0)	8 (40.0)^a,b,d^	23 (63.9)		
	Nonreceptive	48 (31.0)	6 (21.4)	13 (26.0)	4 (19.0)	12 (60.0)^a,b,d^	13 (36.1)		

^a^Different from Profile 1, *P*<.05.

^b^Different from Profile 2, *P*<.05.

^c^n=154.

^d^Different from Profile 3, *P*<.05.

### Receptiveness to IT Use in Physical Activity

Of the 155 participants, a total of 107 (69.0%) responded that they could imagine supplementing exercise with the use of IT solutions. Sociodemographic and disease-related characteristics, mental well-being, smoking habits, and alcohol consumption are presented in Multimedia Appendix 1 for participants that were receptive and nonreceptive to IT use in PA, respectively. Increasing age decreased the odds of being receptive (OR=0.94, 95% CI 0.90-0.97; *P*=.001). There were no significant associations between receptive and nonreceptive participants regarding the remaining exposure variables. Nonreceptive participants scored lower on eHLQ1-using technology to process health information (*P*=.001), eHLQ3-ability to actively engage with digital services (*P*=.001), and eHLQ5-motivated to engage with digital services (*P*=.001) compared to receptive participants (Multimedia Appendix 2).

## Discussion

The main finding of this study is the identification of vulnerable subgroups of individuals with T2D characterized by low mental well-being, emotional distress, and low readiness for health technology. Notably, the findings indicate that these vulnerable subgroups could not be identified by their disease-related and sociodemographic characteristics, including ambiguous findings according to age. Thus, it is crucial that IT-supported T2D health care is individually tailored based on an evaluation of mental well-being, emotional distress, and readiness for health technology rather than sociodemographic characteristics, including age and the severity of T2D.

This is the first study to add measures of self-management and social support to eHealth literacy in a profound understanding of readiness for health technology among individuals with T2D referred to a lifestyle program. The stratification of individuals into profiles based on their level of readiness for health technology is supported by previous findings among individuals with cancer referred to a rehabilitation program [19]. In this study, we identified a subgroup (Profile 3) of relatively younger individuals outside the labor market with a particularly high level of emotional distress, which we do not see among cancer survivors. In the context of diabetes, emotional distress has previously been described as diabetes distress [10]. Diabetes distress may affect up to 45% of individuals with T2D, of which 70% do not meet the criteria for major depressive disorder (MDD) [10]. In line with MDD, individuals with diabetes distress are less likely to engage in self-managing behaviors, which negatively affects health outcomes (for example, leading to poor glycemic control) [10]. The medium level of eHealth literacy along with the high receptiveness to IT use in PA in this subgroup indicates that under the right conditions, IT implementation may be a beneficial alternative or supplement to center-based exercise programs. We report a strong correlation between low mental well-being (an indicator of risk of depression [18]) and emotional distress (an indicator of diabetes distress [10]). Individuals with MDD may benefit from pharmacotherapy, whereas individuals with diabetes distress are not likely to [10]. Diabetes self-management education is an effective treatment for diabetes distress [10]. Therefore, by distinguishing between depression and diabetes distress, self-management education could be a specific focus when implementing IT in the health care of this subgroup.

We identified 2 additional vulnerable subgroups. One of these (Profile 5) was characterized by low mental well-being and low readiness for health technology. Self-reported diagnosis of depression has previously been negatively associated with eHealth literacy [22]. This indicates that if IT is implemented in health care, it should include extensive support, covering social, self-management, and IT-related aspects. The other subgroup (Profile 4) was characterized by older age, higher mental well-being, as well as a lower level of IT ownership, use, and receptiveness. This subgroup had low eHealth literacy and a medium level of self-management and social support. Rossen and colleagues [19] suggest that IT implementation among such subgroups should be based on a dialogue with the individual about the potential benefits of using IT along with a thorough introduction and IT support. For some individuals in both of these subgroups, this support may not be sufficient to prevent treatment failure, and IT support should be implemented with caution.

In contrast to individuals with cancer and a previous South Korean study in T2D, individuals with low versus high readiness for health technology in this study were not characterized by a sociodemographic gradient [19,23]. The South Korean study used a Korean version of the eHealth Literacy Scale (eHEALS) tool [24], which assesses individuals’ combined knowledge, comfort, and perceived skills at finding, evaluating, and applying electronic health information to health problems [24], while in our study, we include measures of self-management and social support in a new understanding of eHealth literacy. This variation in the understanding of eHealth literacy potentially explains the discrepancies between the studies. Moreover, discrepancies may be attributed, in part, to cultural differences in eHealth literacy between Denmark and South Korea. Specifically, smartphone ownership and internet use among the Danish and South Korean populations is ≥86% and ≥90%, respectively, regardless of sociodemographic characteristics [4,25], indicating that differences may primarily be attributed to cultural differences in health literacy [26]. However, none of the disease-related characteristics reported by Kim and colleagues [23] were associated with eHealth literacy, which agrees with the present findings.

Previous studies investigating the association of sociodemographic and disease-related characteristics to eHealth literacy among healthy individuals and individuals with chronic conditions report rather ambiguous findings [22,27-29]. However, these studies generally agree that age is negatively associated with eHealth literacy [22,27,28]. Our findings indicate that younger age may generally be associated with higher odds of being receptive to IT use for physical activity purposes; however, previous findings do not directly support our identification of a relatively younger vulnerable subgroup (Profile 3). This indicates that a thorough assessment of mental well-being and diabetes distress among relatively younger individuals with T2D is warranted before delivering IT-supported health care.

Limitations of this study include the cross-sectional design, which precludes causal inferences regarding the effects of targeting readiness for health technology in health care and the potential mediating effects of socioeconomic status and mental well-being. Longitudinal designs should be implemented to investigate how this stratification of individuals with T2D reflects interindividual health effects of IT-supported health care. This will clarify the need for social support, self-management education, and IT support among different subgroups, and elucidate whether IT-supported health care induces changes in individual levels of readiness for health technology. Further, the sample representativeness may be suboptimal. First, referral to lifestyle programs is potentially limited among individuals with low socioeconomic status [30]. Second, at the time of data collection, the READHY tool was only validated in Danish, precluding participation from non-Danish speaking individuals, such as individuals from ethnic minority groups. As such, the most vulnerable individuals with T2D may not be fully represented in this convenience sample, indicating an underrepresentation of the identified vulnerable subgroups according to magnitude and diversity. This emphasizes the importance of comprehensively considering the need for social support, self-management education, and extensive IT support when implementing IT in health care. Moreover, as participants constituted a convenience sample, no a priori sample size calculation was performed, increasing the risk of false-negative findings (type 2 errors). Finally, with the strong correlation between mental well-being and heiQ8-emotional distress, it is not surprising that profiles with low levels of emotional distress score high on mental well-being, and vice versa. Two profiles scored similarly low on mental well-being; however, interestingly, one of these (Profile 3) was characterized by a particularly high level of emotional distress.

In this study, we identified vulnerable subgroups of individuals with T2D characterized by low mental well-being, emotional distress, and low readiness for health technology, who could not be identified by their sociodemographic and disease-related characteristics. Based on this investigation, we suggest that implementation of IT in the health care of individuals with T2D should be based on a comprehensive consideration of mental well-being, emotional distress, and readiness for health technology to identify the individuals in need of social support, self-management education, and extensive IT support. IT solutions should possibly be tailored to accommodate these needs and should not stand alone. Overall, a one-size-fits-all approach to IT implementation in health care will potentially increase the risk of treatment failure among the most vulnerable individuals with T2D.

## Appendix

Multimedia Appendix 1 Odds ratio for being receptive to IT use in physical activity according to sociodemographic and disease-related characteristics, mental well-being, smoking habits, and alcohol consumption.

Multimedia Appendix 2 Readiness and Enablement Index for Health Technology (READHY) scale scores for participants that are receptive vs. nonreceptive to IT use in physical activity.

## Abbreviations

ANOVA: analysis of variance
eHLQ: eHealth Literacy Questionnaire
heiQ: Health Education Impact Questionnaire
HLQ: Health Literacy Questionnaire
IT: information technology
MDD: major depressive disorder
PA: physical activity
pp: percent points
READHY: Readiness and Enablement Index for Health Technology
T2D: type 2 diabetes
WHO-5: 5-item World Health Organization Well-Being Index
