# Supplementary material for: Health Technology Readiness Profiles Among Danish Individuals With Type 2 Diabetes: Cross-Sectional Study
Source: J Med Internet Res. 2020 Sep 15;22(9):e21195. doi: 10.2196/21195 (PMC7525399; doi:10.2196/21195)
Supplement: Multimedia Appendix 1 [file jmir_v22i9e21195_app1.docx]

**eTable 1:** Odds ratio for being receptive to IT use in physical activity according to sociodemographic and disease-related characteristics, mental well-being, smoking habits, and alcohol consumption

|  | Receptive | Non-receptive | Odds ratio (95% CI) | *P* value |
| --- | --- | --- | --- | --- |
| n (%) | 107 (69.0) | 48 (31.0) |  |  |
| *Sociodemographic factors* |  |  |  |  |
| Sex |  |  |  | .73 |
| Women, n (%) | 49 (45.8) | 20 (41.7) | 1.00 (reference) |  |
| Men, n (%) | 58 (54.2) | 28 (58.3) | 0.85 [0.33; 2.17] |  |
| Age, mean (SD) | 58.2 (9.8) | 64.8 (11.2) | 0.94 [0.90; 0.97] | <.001 |
| Highest attained level of education |  |  |  | .43 |
| Comprehensive school, n (%) | 15 (14.0) | 9 (18.8) | 1.00 (reference) |  |
| Short education, n (%) | 53 (49.5) | 28 (58.3) | 1.14 [0.83; 1.55] |  |
| Medium education, n (%) | 26 (24.3) | 7 (14.6) | 2.23 [0.31; 16.10] |  |
| Long education, n (%) | 13 (12.2) | 4 (8.3) | 1.95 [0.38; 10.13] |  |
| Cohabitation status |  |  |  | .73 |
| Living alone, n (%) | 55 (51.4) | 23 (47.9) | 1.00 (reference) |  |
| Living with spouse and/or children, n (%) | 52 (48.6) | 25 (52.1) | 0.87 [0.39; 1.92] |  |
| Source of income |  |  |  | .45 |
| Salary, n (%) | 34 (31.8) | 11 (22.9) | 1.00 (reference) |  |
| Retirement pension, n (%) | 47 (43.9) | 26 (54.2) | 0.58 [0.14; 2.38] |  |
| Public income support or no income, n (%) | 26 (24.3) | 11 (22.9) | 0.76 [0.38; 1.54] |  |
| *Disease-related characteristics* |  |  |  |  |
| Time since T2D diagnosis |  |  |  | 1.00 |
| ≤5 years, n (%) | 85 (79.4) | 39 (81.2) | 1.00 (reference) |  |
| ≥6 years, n (%) | 22 (20.6) | 9 (18.8) | 1.12 [NE] |  |
| Type of medication |  |  |  | .31 |
| None, n (%) | 16 (14.9) | 3 (6.3) | 1.00 (reference) |  |
| Peroral, n (%) | 69 (64.5) | 33 (68.7) | 0.39 [0.06; 2.39] |  |
| Injection, n (%) | 22 (20.6) | 12 (25.0) | 0.34 [0.04; 2.70] |  |
| T2D complications^†^ |  |  |  | .65 |
| No, n (%) | 66 (75.0) | 24 (70.6) | 1.00 (reference) |  |
| Yes, n (%) | 22 (25.0) | 10 (29.4) | 0.80 [0.31; 2.10] |  |
| Additional chronic conditions |  |  |  | .29 |
| no additional conditions, n (%) | 13 (12.1) | 4 (8.3) | 1.00 (reference) |  |
| 1 additional condition, n (%) | 37 (34.6) | 23 (47.9) | 0.49 [0.13; 1.83] |  |
| 2+ additional conditions, n (%) | 57 (53.3) | 21 (43.8) | 0.84 [0.60; 1.17] |  |
| Mental well-being, mean (SD)^‡^ | 58.0 (18.6) | 61.8 (23.6) | 0.99 [0.97; 1.01] | .28 |
| Risk of depression (score <50), n (%)^‡^ |  |  |  | 1.00 |
| No, n (%) | 73 (68.2) | 32 (68.1) | 1.00 (reference) |  |
| Yes, n (%) | 34 (31.8) | 15 (31.9) | 1.02 [NE] |  |
| *Lifestyle factors* |  |  |  |  |
| Smoking habits^‡^ |  |  |  | .35 |
| Current, n (%) | 18 (17.0) | 8 (16.6) | 1.00 (reference) |  |
| Earlier, n (%) | 56 (52.8) | 20 (41.7) | 1.24 [0.79; 1.97] |  |
| Never, n (%) | 32 (30.2) | 20 (41.7) | 0.71 [0.35; 1.46] |  |
| Alcohol consumption^‡^ |  |  |  | .22 |
| No alcohol, n (%) | 34 (32.1) | 21 (43.7) | 1.00 (reference) |  |
| According to recommendations, n (%) | 60 (56.6) | 25 (52.1) | 1.48 [0.80; 2.76] |  |
| Above recommendations, n (%) | 12 (11.3) | 2 (4.2) | 3.71 [0.47; 29.39] |  |

Data are presented as mean (SD) for continuous variables and numbers (proportions) for frequencies. NE=non-estimable. ^†^n=122; ^‡^n=154
