# Supplementary material for: Health Technology Readiness Profiles Among Danish Individuals With Type 2 Diabetes: Cross-Sectional Study
Source: J Med Internet Res. 2020 Sep 15;22(9):e21195. doi: 10.2196/21195 (PMC7525399; doi:10.2196/21195)
Supplement: Multimedia Appendix 2 [file jmir_v22i9e21195_app2.docx]

**eTable 2:** READHY scale scores for participants that are receptive vs. non-receptive to IT use in physical activity

|  | Receptive | Non-receptive | F value | *P* value |
| --- | --- | --- | --- | --- |
| heiQ3 Self-monitoring and insight | 2.99 (0.49) | 2.88 (0.46) | 1.91 | .17 |
| heiQ4 Constructive attitudes and approaches | 2.98 (0.62) | 2.98 (0.65) | 0.00 | .94 |
| heiQ5 Skills and technique acquisition | 2.87 (0.59) | 2.93 (0.58) | 0.32 | .57 |
| heiQ8 Emotional distress^†^ | 2.58 (0.75) | 2.74 (0.77) | 1.62 | .20 |
| HLQ1 Feeling understood and supported by healthcare providers | 3.03 (0.62) | 3.06 (0.53) | 0.08 | .77 |
| HLQ4 Social support for health | 2.77 (0.71) | 2.85 (0.67) | 0.39 | .53 |
| eHLQ1 Using technology to process health information | 2.70 (0.64) | 2.26 (0.55) | 16.92 | <.001 |
| eHLQ2 Understanding of health concepts and language | 3.00 (0.49) | 2.88 (0.50) | 2.04 | .16 |
| eHLQ3 Ability to actively engage with digital services | 2.96 (0.64) | 2.42 (0.65) | 23.31 | <.001 |
| eHLQ4 Feel safe and in control | 2.94 (0.56) | 2.93 (0.46) | 0.02 | .90 |
| eHLQ5 Motivated to engage with digital services | 2.69 (0.59) | 2.34 (0.52) | 12.94 | <.001 |
| eHLQ6 Access to digital services that work | 2.71 (0.53) | 2.61 (0.47) | 1.19 | .28 |
| eHLQ7 Digital services that suit individual needs | 2.56 (0.59) | 2.38 (0.58) | 3.42 | .07 |

Data are presented as mean (SD). READHY=Readiness and Enablement Index for Health Technology; heiQ=health education impact Questionnaire; HLQ=Health Literacy Questionnaire; eHLQ=eHealth Literacy Questionnaire. The *heiQ8* scale was reversed, i.e. a high score indicates a low level of emotional distress.
